# Comparative Analysis of *FCGR* Gene Polymorphism in Pulmonary Sarcoidosis and Tuberculosis

**DOI:** 10.3390/cells12091221

**Published:** 2023-04-23

**Authors:** Marlena Typiak, Bartłomiej Rękawiecki, Krzysztof Rębała, Anna Dubaniewicz

**Affiliations:** 1 Department of General and Medical Biochemistry, Faculty of Biology, University of Gdansk, 80-308 Gdansk, Poland; marlena.typiak@ug.edu.pl; 2 Department of Pulmonology, Medical University of Gdansk, 80-214 Gdansk, Poland; 3 Department of Forensic Medicine, Medical University of Gdansk, 80-204 Gdansk, Poland

**Keywords:** sarcoidosis, tuberculosis, Fc gamma receptors, IgG receptors, gene polymorphism, *FCGR* polymorphism, single nucleotide polymorphism, SNPs

## Abstract

The clinical outcome of sarcoidosis (SA) is very similar to tuberculosis (TB); however, they are treated differently and should not be confused. In search for their biomarkers, we have previously revealed changes in the phagocytic activity of monocytes in sarcoidosis and tuberculosis. On these monocytes we found a higher expression of receptors for the Fc fragment of immunoglobulin G (FcγR) in SA and TB patients vs. healthy controls. FcγRs are responsible for the binding of immune complexes (ICs) to initiate an (auto)immune response and for ICs clearance. Surprisingly, our SA patients had a high blood level of ICs, despite the abundant presence of FcγRs. It pointed to FcγR disfunction, presumably caused by the polymorphism of their (*FCGR)* genes. Therefore, we present here an analysis of the occurrence of *FCGR2A*, *FCGR2B*, *FCGR2C*, *FCGR3A* and *FCGR3B* variants in Caucasian SA and TB patients, and healthy individuals with the use of polymerase chain reaction (PCR) and real-time PCR. The presented data point to a possibility of supporting the differential diagnosis of SA and TB by analyzing *FCGR2C*, *FCGR3A* and *FCGR3B* polymorphism, while for severe stages of SA also by studying *FCGR2A* variants. Additionally, the genotyping of *FCGR2A* and *FCGR3B* might serve as a marker of SA progression.

## 1. Introduction

Sarcoidosis (SA) is a granulomatous disease with an unknown aetiology. Genetics, environmental factors and autoimmunity are considered in the etiopathogenesis of SA [1,2,3,4,5]. Due to similar clinical, radiological and histopathological manifestations of SA and tuberculosis (TB), mycobacterial antigens have often been tested in sarcoidosis [1,2,3,5,6]. Because of these similarities between the two diseases, a misdiagnosis can occur. The differentiation between SA and TB is of great importance, since immunosuppressive treatment, used in sarcoidosis, can have very dangerous outcomes during active TB. Our team has also previously reported cases of patients, primarily diagnosed with sarcoidosis, who have developed TB after immunosuppressive treatment [1,4,5,6].

Our team has previously found a presence of *M. tuberculosis* heat shock proteins (Mtb-HSP70, Mtb-HSP65 and Mtb-HSP16) in the lymph nodes of patients diagnosed with sarcoidosis [7,8,9]. In the serum of the same SA patients, an increased level of immunoglobulin G (IgG)-based immune complexes (ICs) with Mtb-HSP was revealed. The most abundant Mtb-HSP protein in these ICs was Mtb-HSP16; a marker of a dormant stage of tuberculosis [7,8,10]. The immunocomplexemia in the blood of SA patients was especially present during the initial stages of the disease. Moreover, the level of ICs with Mtb-HSP16 was higher in SA patients than in patients suffering from tuberculosis [8,10].

Because immunocomplexemia can be a result of an abnormal function of the receptors for Fc fragment of IgG (FcγRs), especially receptors belonging to Class II and III, we evaluated the fraction of FcγRI^+^ (CD64), FcγRII^+^ (CD32) and FcγRIII^+^ (CD16) blood monocytes in the same patients with SA and TB [11]. The increased frequency of FcγRI^+^, FcγRII^+^ and FcγRIII^+^ monocytes was revealed in SA, whereas increase in the fraction of monocytes with FcγR Class II was found in TB [12,13,14].

Therefore, in the context of an abundant number of blood phagocytes possessing FcγRs, but not eliminating ICs from circulation, we hypothesised that there receptors in SA and TB had a possible malfunction. This aberrant function may be caused by a functional polymorphism of genes (*FCGR* genes) encoding FcγRs, which we have already shown in part regarding sarcoidosis versus healthy individuals [12,13]. The differences between *FCGR* gene polymorphism in SA and TB could help us understand the etiopathogeneses of both sarcoidosis and tuberculosis, pointing to their similarities and/or differences. Additionally, up to now, there have been no published studies on *FCGR* gene polymorphism in TB patients in the Caucasian ethnic group.

Therefore, the aim of this work was to assess, if certain functional polymorphisms of the genes that encode the activating FcγRs (*FCGR2A* encoding FcγRIIa, *FCGR2C*—FcγRIIc, *FCGR3A*—FcγRIIIa, *FCGR3B*—FcγRIIIb) and/or inhibiting FcγRIIb (*FCGR2B* gene) can predispose SA and TB development. In addition, this study could identify a molecular marker that may help differentiate between the two diseases and support the process of their diagnosis.

## 2. Materials and Methods

### 2.1. Characteristics of the Studied Groups

The material, for gene variants analysis, consisted of peripheral blood samples, collected from patients diagnosed with sarcoidosis, patients with tuberculosis and healthy volunteers. Individuals from northern Poland, of Caucasian ethnicity, were enrolled in the study. The characteristics (including clinical data) of the studied groups are presented in Table 1.

### 2.2. Patients with Sarcoidosis

The study enrolled 154 unrelated sarcoidosis patients, who were under the care of clinics for lung diseases, located in the Gdansk–Sopot–Gdynia city agglomeration (Poland) in the years 2013–2020. Confirmation of the diagnosis of sarcoidosis in these patients was made on the basis of clinical symptoms and radiological and histopathological examinations with confirmation of the presence of noncaseating epithelioid cell granulomas in the lymph node biopsy. The diagnosis was performed in accordance with criteria, stated jointly by the American Thoracic Society, the European Respiratory Society and the World Association of Sarcoidosis and Other Granulomatous Disorders [1]. Sputum and biopsy material taken from the patients did not reveal acid-fast bacilli (PCR, M. tuberculosis culture), fungal infection or atypical cells. An additional criterion for inclusion in the study for SA patients was a negative PPD skin test.

Some of the SA patients presented general (e.g., fever) and/or organ-specific symptoms for less than two years (acute form of SA), with of without symptom characteristic for Löfgren’s syndrome. Manifestations of Löfgren’s syndrome include: erythema nodosum (painful, large, reddened infiltrates in the subcutaneous tissue, localized mostly on the front side of the lower legs), hilar lymphadenopathy (enlargement of the lymph nodes in the cavities of the lungs), swelling of ankles and/or elevated temperature. Patients with SA symptoms, such as cough, weight loss, cardiopulmonary insufficiency or a malfunction of another organ due to granuloma formation, lasting more than two years from the time of the onset of the disease, were diagnosed with a chronic type of sarcoidosis [15]. Twelve patients with SA (8%) experienced a loss of body weight.

Five radiological stages of sarcoidosis have been differentiated on the basis of the Scadding system. Stage 0 of SA is equal to a normal chest radiograph, and points to the existence of extrapulmonary SA. In Stage I of SA, bilateral hilar lymphadenopathy is present with no changes in the lungs. In Stage II of SA, bilateral hilar lymphadenopathy is accompanied by early changes in the lung parenchyma. In Stage III of SA, nodules occur only in the parenchyma. The development of the disease to Stage IV is connected with irreversible pulmonary fibrosis and severe malfunction of the respiratory system [2]. The early stages (I or II) of pulmonary sarcoidosis can be followed by advanced stages (III or IV), in which only the lungs (and not the lymph nodes) are affected by the disease. The numbers of patients in particular stages of the disease are included in Table 2.

### 2.3. Patients with Tuberculosis

In the study, 179 unrelated patients with active pulmonary tuberculosis were included before the initiation of antituberculosis therapy in these individuals. The patients were hospitalized at the Pomeranian Center for Infectious Diseases and Tuberculosis in Gdansk (Poland) and in nearby facilities. The diagnosis of tuberculosis was made on the basis of the clinical, radiological picture and, above all, a positive bacteriological test result for the presence of *Mycobacterium tuberculosis* in the sputum (bacterioscopy and culture). A positive PPD skin test (reaction > 10 mm) was an additional criterion for inclusion in the study. Forty-eight patients with TB (27%) showed a loss of body weight due to the disease development.

### 2.4. Healthy Control

The reference group consisted of 148 healthy unrelated volunteers from northern Poland (Gdansk city and its close proximity) who had no family history of tuberculosis, sarcoidosis or other autoimmune diseases. The healthy controls did not experience a loss of body weight.

### 2.5. Sample Collection and DNA Isolation

Small (10 mL) peripheral blood samples were collected from the participants of the study, as previously described [12]. Genomic DNA was isolated from those samples, as described by Lahiri and Nurnberger [16].

### 2.6. Polymerase Chain Reaction with Sequence-Specific Primers

A polymerase chain reaction (PCR) with sequence-specific primers (PCR-SSP) was used to study the functional polymorphism of *FCGR2A, FCGR2C, FCGR3A* and *FCGR3B* genes. The designation of the exact polymorphic sites, that were analyzed, and the sequences of the used primers are shown in Table 3. A very detailed description of the used methods is presented in our previous articles, regarding the polymorphism of these genes in sarcoidosis, in comparison to healthy individuals [12,13].

Separate PCR reactions were performed for each (of the two) variants of *FCGR2A*, *FCGR2C* and *FCGR3A* genes, due to the exact same length of the PCR products corresponding to that variants. To exclude false negative results in these PCR reactions, an internal control of amplification was added to the PCR mixtures. Thus, primers for the human growth hormone (*hGH*) gene fragment were added to the PCR mixtures for the *FCGR2A* and *FCGR2C* gene variants, and primers for a fragment of the neurofibromin 1 (*NF1*) gene were added to the mixture for studying *FCGR3A* alleles (*NF1* exon 4b for 158F allele; *NF1* exon 22 for 158V allele). One joint PCR mixture was prepared for studying three alleles of the *FCGR3B* gene; it did not need an internal control of amplification.

The primer 3B-NA2/SH and 3B-gene used in the study are substrates for the creation of a product specific both to NA2 and the SH alleles (NA2/SH product) in the PCR reaction. Supplementing the PCR-SSP reaction with the primer 3B-SH allows us to obtain a product specific only to the SH allele, and distinguish between the NA1/NA2 and NA1/SH genotypes. In the case of the simultaneous presence of PCR products specific both to the NA2/SH and SH alleles; the method used cannot distinguish between NA2/SH and SH/SH genotypes. Due to the significantly lower occurrence of the SH allele than the NA2 allele in the Caucasian population (10% vs. 63%), the obtained result was interpreted as the presence of the NA2/SH genotype.

### 2.7. Real-Time Polymerase Chain Reaction

Due to a difficulty in studying the allelic variants of the *FCGR2B* gene, which is very similar in sequence to the *FCGR2C* gene, we have decided to use the real-time PCR method to study the polymorphism of *FCGR2B*. We have used a custom-made (Assays-by-Design service of Applied Biosystems, Foster City, USA) Custom Taqman SNP Genotyping Assay. The assay consisted of a TaqMan Genotyping Master Mix (2×) and TaqMan SNP Genotyping Assay (40×), which included primers for PCR reaction and two probes with different fluorescent dyes (VIC for 695T and FAM for 695C allele). The primers were common for both *FCGR2B* gene variants. The gene variants were differentiated based on the hybridisation of a variant-specific probe (Table 4). Experiments were performed with 7900HT Fast Real-Time PCR System (Applied Biosystems) in 384-well plates. In each test, a well 2 µL (5 ng/µL) DNA solution was placed and dried in room temperature. Subsequently, 5 µL of a reaction mix was added to the well. The reaction mix consisted of TaqMan Genotyping Master Mix and TaqMan SNP Genotyping Assay (both diluted in water to 1× concentration). The temperature profile of the reaction comprised of 10 min in 95 °C and 50 amplification cycles (15 s 95 °C, 1 min 60 °C).

### 2.8. Electrophoresis and DNA Staining

The electrophoresis and DNA staining within the polyacrylamide gel was performed as described before [13].

### 2.9. Statistical Analysis

The STATISTICA v.13 for Windows (StatSoft, Tulsa, OK, USA) programme was used to assess if the study groups were properly selected in terms of age and gender distribution and to analyse the obtained data. Datasets were checked for their adherence to the conditions of a normal distribution (Shapiro–Wilk test). The age of people in the study groups was compared using the Mann–Whitney U test. The Fisher test was implemented to compare the number of women and men, as well as allele and genotype frequencies in the studied groups. A significant result of comparison was considered for a *p* value lower than 0.05. Significant differences were presented in full form.

Odds ratios (OR) and their 95% confidence intervals (CIs) were calculated to assess the risk of developing SA and TB. ORs and CIs for certain alleles were calculated using the Vassar Stats program [22]. ORs and CIs for certain genotypes were calculated using the OR calculator OEGE program [23]. The Hardy–Weinberg equilibrium calculator program (developed by Rodriguez et al.) was used to verify the compatibility of the obtained genotype frequencies with the Hardy–Weinberg law [24].

## 3. Results

The studied groups were consistent in terms of age (SA vs. healthy control: *p* = 0.52; SA vs. TB: *p* = 0.57; TB vs. healthy control: *p* = 0.08) and sex (SA vs. healthy control: *p* = 0.97; SA vs. TB: *p* = 0.29; TB vs. healthy control: *p* = 0.97).

The comparison of the frequency of *FCGR2A*, *FCGR2C*, *FCGR3A* and *FCGR3B* gene variants in the same SA patient group and healthy individuals has been previously published by our team [12,13]. Thus, in the current work, we will present the comparison of the frequencies of these gene variants between SA and TB patients, as well as between TB patients and healthy controls, which has never been shown before for the Caucasian ethnic group.

### 3.1. FCGR2A Gene Polymorphism

The distribution of the number of certain genotypes of *FCGR2A* was consistent with the Hardy–Weinberg law in the group of patients with SA (*p* = 0.87), TB (*p* = 0.55) and in the group of healthy individuals (*p* < 0.99). The results of the *FCGR2A* polymorphism were established for 144 SA patients (36 in Stage I, 53—Stage II, 34—Stage III, 21—Stage IV of SA), 179 TB patients and 145 healthy individuals.

**Figure 1:**
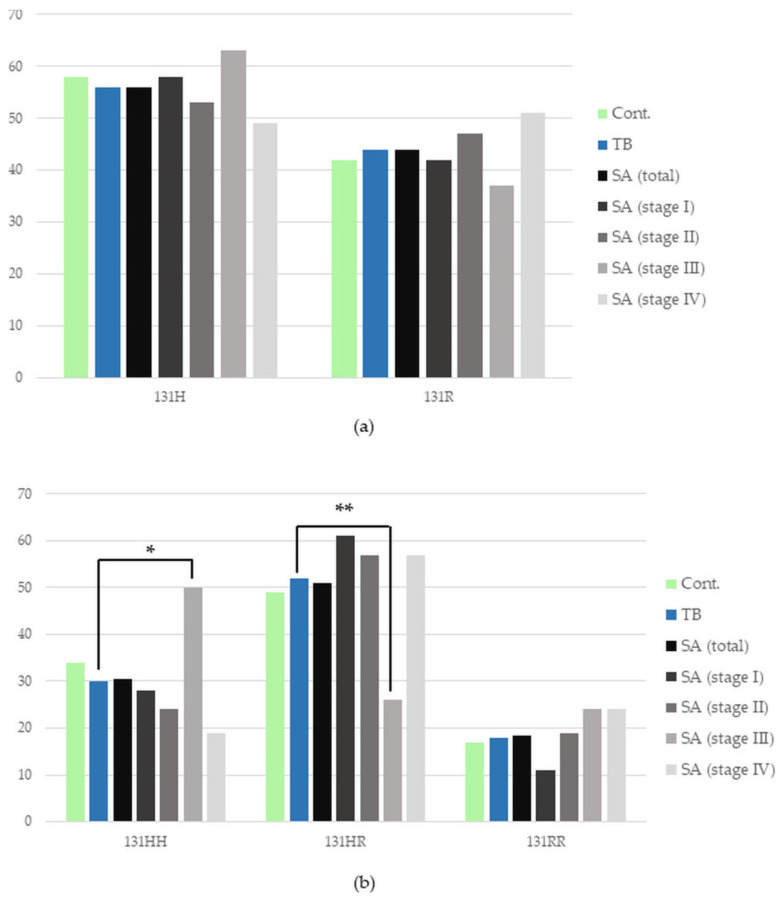
Frequency of the occurrence of individual (**a**) alleles and (**b**) genotypes of the *FCGR2A* gene in the studied groups and subgroups. Cont.—healthy control; SA—group of patients with sarcoidosis; TB—group of patients with tuberculosis; * *p* = 0.03; ** *p* = 0.008.

There was no statistical difference in the frequency of the presence of *FCGR2A* alleles or genotypes between the whole group of SA and TB patients, as well as between TB patients and the control group (*p* > 0.05). There were also no statistically significant differences in the percentage of *FCGR2A* alleles between patients in various stages of sarcoidosis and patients with pulmonary tuberculosis (*p* > 0.05). However, a significantly higher incidence of the 131HH genotype and a significantly lower incidence of the 131HR genotype in Stage III sarcoidosis compared to patients with TB were found (50% vs. 30%, respectively, *p* = 0.03, OR = 2.38, CIs 1.13–5.01, 26% vs. 52%, *p* = 0.008, OR = 0.33, CIs 0.15–0.75) (Figure 1). The detailed numbers and percentages of certain alleles and genotypes of the *FCGR2A* gene in the studied groups are presented in Tables S1 and S2.

### 3.2. FCGR2B Gene Polymorphism

The distribution of the number of certain genotypes of *FCGR2B* was consistent with the Hardy–Weinberg law in the group of patients with SA (*p* = 0.70) and TB (*p* = 0.20), and in the group of healthy individuals (*p* = 0.69). The results of the *FCGR2B* polymorphism were established for 134 SA patients (30 in Stage I, 49—Stage II, 34—Stage III, 21—Stage IV of SA), 97 TB patients and 101 healthy individuals.

There was no statistical difference in the frequency of the presence of *FCGR2B* alleles or genotypes between SA (whole SA group and certain stages of SA) and TB patients, as well as between TB patients and the control group (*p* > 0.05). The detailed numbers and percentages of certain alleles and genotypes of the *FCGR2B* gene are presented in Tables S3 and S4.

### 3.3. FCGR2C Gene Polymorphism

The distribution of the number of certain genotypes of *FCGR2C* was consistent with the Hardy–Weinberg law in the group of patients with SA (*p* = 0.11) and in healthy individuals (*p* = 0.59), but was not within the group of patients with TB (*p* = 0.001). The results of the *FCGR2C* polymorphism were established for 149 SA patients (37 in Stage I, 57—Stage II, 34—Stage III, 21—Stage IV of SA), 179 TB patients and 148 healthy individuals.

#### 3.3.1. *FCGR2C* Polymorphism in the Whole SA Group vs. TB, and in TB vs. Control

**Figure 2:**
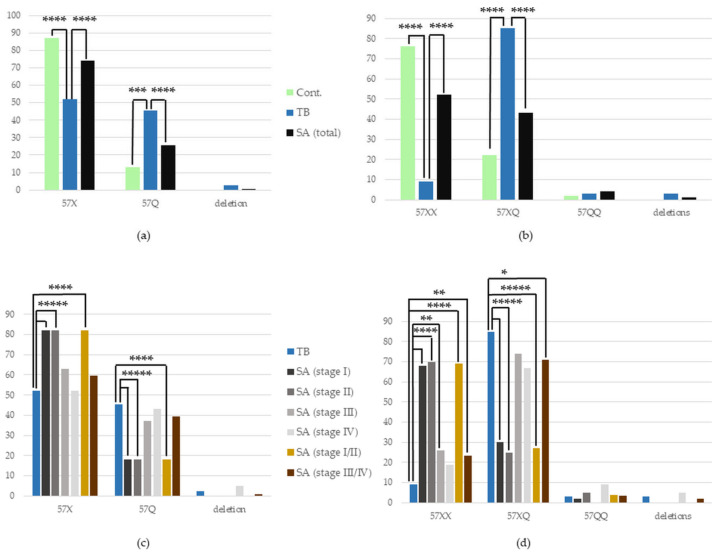
Frequency of the occurrence of individual (**a**,**c**) alleles and (**b**,**d**) genotypes of the *FCGR2C* gene in the studied (**a**,**b**) groups and (**c**,**d**) subgroups. Cont.—healthy control; SA—group of patients with sarcoidosis; TB—group of patients with tuberculosis; * *p* < 0.05, ** *p* < 0.01, *** *p* < 0.001, **** *p* < 0.0001, ***** *p* < 0.00001.

We have found a higher occurrence of the 57X in the patients with SA and in healthy individuals (Cont.) compared to those with TB (SA vs. TB: 74% vs. 52%, *p* < 0.0001, OR = 2.58, CIs 1.85–3.59, TB vs. Cont.: 52% vs. 87%, *p* < 0.0001, OR = 0.17, CIs 0.12–0.26). We have also found a lower frequency of the 57Q allele in SA patients and the control compared with TB patients (SA vs. TB—25.5% vs. 45.5%, *p* < 0.0001, OR = 0.41, CIs 0.29–0.57; TB vs. Cont.—45.5% vs. 13%, *p* < 0.001, OR = 5.74, CIs 3.86–8.54) (Figure 2). This aberration in the allele distribution of the *FCGR2C* gene also translated into the changed frequency of the *FCGR2C* genotypes.

The 57XX genotype occurred significantly more often in SA patients and healthy individuals than in the group of TB patients (SA vs. TB—52% vs. 9%, *p* < 0.0001, OR = 10.47, CIs 5.78–18.96, TB vs. Cont.—9% vs. 76%, *p* < 0.0001, OR = 0.03, CIs 0.02–0.06). There was also a less frequent occurrence of the 57XQ genotype in SA patients and healthy individuals than in TB patients (SA vs. TB: 43% vs. 85%, *p* < 0.0001, OR = 0.13, CIs 0.08–0.22; TB vs. Cont.: 85% vs. 22%, *p* < 0.0001, OR = 20.51, CIs 11.62–36.19) (Figure 2). The detailed numbers and percentages of certain alleles and genotypes of the *FCGR2C* gene in the studied groups are presented in Tables S5 and S6.

#### 3.3.2. *FCGR2C* Polymorphism in Certain Stages of SA vs. TB

A significantly more frequent occurrence of the 57X allele was found in SA patients in Stages I and II of the disease, in comparison to TB patients (SA Stage I vs. TB: 82% vs. 52%, *p* < 0.00001, OR = 4.09, CIs 2.17–7.71; SA Stage II vs. TB: 82% vs. 52%, *p* < 0.00001, OR = 4.10, CIs 2.42–6.93). Complementary to this, there was a lower presence of the 57Q allele in the group of patients with SA in Stage I and II than in the TB group (SA Stage I vs. TB: 18% vs. 45.5%, *p* < 0.00001. OR = 0.24, CIs 0.13–0.46; SA Stage II vs. TB: 18% vs. 45.5%, *p* < 0.00001, OR = 0.24, CIs 0.14–0.41). In the joined group of patients with SA in Stage I or II (the initial stages of sarcoidosis) versus the TB group, these associations were repeated (57X allele: 82% vs. 52%, *p* < 0.0001, OR = 4.09, CIs 2.66–6.30; 57Q allele: 18% vs. 45.5%, *p* < 0.0001, OR = 0.24, CIs 0.16–0.38, respectively) (Figure 2).

Regarding the genotypes of the *FCGR2C* gene, there was a significant increase in the frequency of the 57XX genotype occurrence in patients with sarcoidosis in Stages I, II and III, in comparison to TB patients (68% vs. 9%, *p* < 0.00001, OR = 19.85, CIs 8.48–46.48; 70% vs. 9%, *p* < 0.0001, OR = 22.42, CIs 10.53–47.76; 26% vs. 9%, *p* = 0.01, OR = 3.43, CIs 1.38–8.53, respectively). We have also found a lower occurrence of the 57XQ genotype in patients with SA in Stages I and II compared with TB patients (30% vs. 85%, *p* < 0.00001, OR = 0.07, CIs 0.03–0.16; 25% vs. 85%, *p* < 0.00001, OR = 0.15, CIs 0.07–0,31, respectively). These associations were repeated in the joined group of patients with SA in Stages I or II versus the TB group (57XX allele: 69% vs. 9%, *p* < 0.0001, OR = 21.36, CIs 10.99–41.50; 57XQ allele: 27% vs. 85% *p* < 0.00001, OR = 0.06, CIs 0.03–0.11). Additionally, in the joint group of patients with more advanced stages of sarcoidosis (Stage III with IV), the 57XX genotype was more frequent, and 57XQ less frequent than in the TB group (24% vs. 9%, *p* = 0.01, OR = 2.95, CIs 1.33–6.55; 46% vs. 85%, *p* = 0.03, OR = 0.41, CIs 0.20–0.85, respectively). The detailed numbers and percentages of certain alleles and genotypes of the *FCGR2C* gene in the studied groups are presented in Tables S5 and S6.

### 3.4. FCGR3A Gene Polymorphism

The analysis of the *FCGR3A* gene polymorphism was the most promising for finding a distinction between the immune response mechanisms, present in sarcoidosis and tuberculosis, due to the previously revealed increase in the percentage of blood monocytes with FcγRIII in SA versus TB [25]. Monocytes have a FcγRIIIa receptor on their surface (encoded by *FCGR3A*), and not the other Class III Fc receptor—FcγRIIIb (encoded by *FCGR3B*) [14].

The distribution of the number of certain genotypes of *FCGR3A* was consistent with the Hardy–Weinberg law in the group of patients with SA (*p* = 0.11), TB (*p* = 0.45) and in healthy individuals (*p* = 0.59). The results of *FCGR3A* polymorphism were established for 120 SA patients (23 in Stage I, 54—Stage II, 23—Stage III, 20—Stage IV of SA), 179 TB patients and 148 healthy individuals.

#### 3.4.1. *FCGR3A* Polymorphism in the Whole SA Group vs. TB, and in TB vs. Control

**Figure 3:**
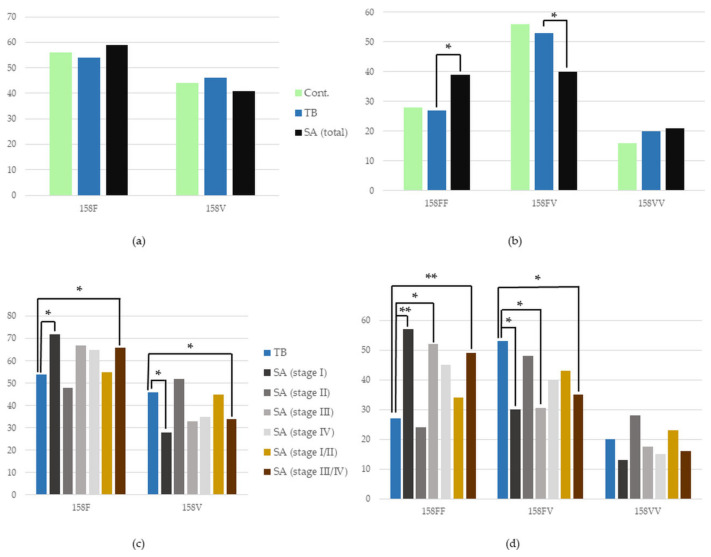
Frequency of the occurrence of individual (**a**,**c**) alleles and (**b**,**d**) genotypes of the *FCGR3A* gene in the studied (**a**,**b**) groups and (**c**,**d**) subgroups. Cont.—healthy control; SA—group of patients with sarcoidosis; TB—group of patients with tuberculosis; * *p* < 0.05, ** *p* ≤ 0.01.

There was no statistical difference in the frequency of the presence of *FCGR3A* alleles between the whole group of SA and TB patients, as well as between TB patients and the control group (*p* > 0.05). However, we found a significant increase in the frequency of the 158FF genotype in the group of patients with SA in comparison to TB patients (39% vs. 27%, *p* = 0.04, OR = 1.71, CIs 1.04–2.79). Additionally, a lower occurrence of the 158FV genotype was shown in patients with SA versus those with TB (40% vs. 53%, *p* = 0.03, OR = 0.59, Cis 0.37–0.94) (Figure 3). The detailed numbers and percentages of certain alleles and genotypes of the *FCGR3A* gene in the studied groups are presented in Tables S7 and S8.

#### 3.4.2. *FCGR3A* Polymorphism in Certain Stages of SA vs. TB

In the initial, Stage I, of pulmonary sarcoidosis, we showed a more frequent occurrence of 158F and a less frequent occurrence of the 158V allele, in comparison to TB patients (72% vs. 54%, *p* = 0.03, OR = 2.17, CIs 1.11–4.26; 28% vs. 46%, *p* = 0.03, OR = 0.46, CIs 0.23–0.90, respectively). In patients with this stage of SA (Stage I), as well as in patients with a more advanced stage, Stage III, versus in patience with TB, an increased presence of 158FF and a decreased presence of the 158FV genotype were found (158FF: 57% vs. 27%, *p* = 0.01, OR = 3.45, Cis 1.42–8.38; 52% vs. 27%, *p* = 0.03, OR = 2.89, Cis 1.20–6.99; 158FV: 30% vs. 53%, *p* = 0.048, OR = 0.39, Cis 0.15–0.99; 30.5% vs. 53%, *p* = 0.048, OR = 0.39, Cis 0.15–0.99, respectively).

In the joint group of patients in the most advanced stages of SA, Stages III and IV, we have also found an increased frequency of 158F and a lowered frequency of the 158V allele compared to patients suffering from TB (66% vs. 54%, *p* = 0.04, OR = 1.68, CIs 1.03–2.75; 34% vs. 46%, *p* = 0.04, OR = 0.60, CIs 0.36–0.97, respectively). It translated to a higher occurrence of 158FF and a lower presence of the 158FV genotype in this joint group of SA versus in TB patients (49% vs. 27%, *p* = 0.01, OR = 2.53, CIs 1.28–5.01; 35% vs. 53%, *p* = 0.02, OR = 0.47, CIs 0.24–0.95, respectively) (Figure 3). The detailed numbers and percentages of certain alleles and genotypes of the *FCGR3A* gene in the studied groups are presented in Tables S7 and S8.

### 3.5. FCGR3B Gene Polymorphism

The distribution of the number of certain genotypes of *FCGR3B* was consistent with the Hardy–Weinberg law in the group of patients with SA (*p* = 0.13) and in healthy individuals (*p* = 0.13), but not in TB patients (*p* < 0.05). The results of the *FCGR3B* polymorphism were established for 148 SA patients (38 in Stage I, 60—Stage II, 35—Stage III, 21—Stage IV of SA), 179 TB patients and 154 healthy individuals.

#### 3.5.1. *FCGR3B* Polymorphism in the Whole SA Group vs. TB, and in TB vs. Control

**Figure 4:**
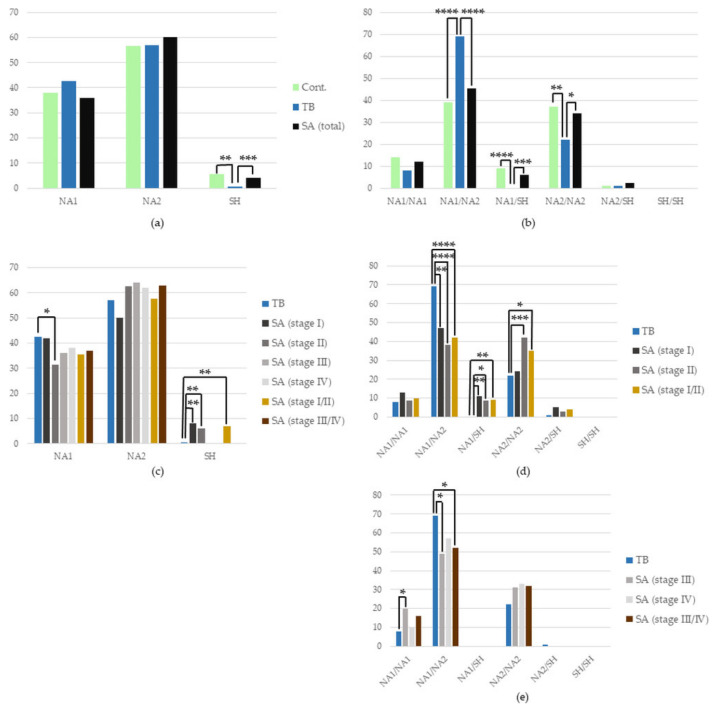
Frequency of the occurrence of individual (**a**,**c**) alleles and (**b**,**d**,**e**) genotypes of the *FCGR3B* gene in the studied (**a**,**b**) groups and (**c**,**d**,**e**) subgroups. Cont.—healthy control; SA—group of patients with sarcoidosis; TB—group of patients with tuberculosis; * *p* ≤ 0.05, ** *p* ≤ 0.01, *** *p* ≤ 0.001, **** *p* ≤ 0.0001.

A more frequent occurrence of the SH allele was found in SA patients and healthy controls than in TB patients (SA vs. TB: 4% vs. 0.5%, *p* = 0.001, OR = 15.73, CIs 2.05–120.97; TB vs. Cont.: 0.5% vs. 5.5%, *p* = 0.004, OR = 0.05, CIs 0.01–0.40). It translated into a higher occurrence of the NA1/SH genotype, possessing the SH allele in the SA group and healthy group versus the TB group (SA vs. TB: 6% vs. 0%, *p* = 0.001, OR = ∞, CIs 0–∞; TB vs. Cont.: 0% vs. 9%, *p* < 0.0001, OR = 0.03, CIs 0.002–0.44). Additionally, in the SA patient and healthy groups, versus the TB patient group, there was an increase in the NA2/NA2 presence, a genotype made from alleles identical by 83.5% with the SH allele (SA vs. TB: 34% vs. 22%, *p* = 0.03, OR = 1.77, CIs 1.09–2.88; TB vs. Cont.: 22% vs. 37%, *p* = 0.003, OR = 0.49, CIs 0.30–0.79). Supplementary to this, in SA patient and healthy groups, compared to TB patients, there was a decreased frequency of the NA1/NA2 genotype occurrence (SA vs. TB: 45.5% vs. 69%, *p* < 0.0001, OR = 0.37, CIs 0.24–0.58; TB vs. Cont.: 69% vs. 39%, *p* < 0.0001, OR = 3.60, CIs 2.28–5.69) (Figure 4).

#### 3.5.2. *FCGR3B* Polymorphism in Certain Stages of SA vs. TB

A higher incidence of the SH allele was shown in SA patients in the initial stages, Stages I and II, of the disease, and in a joint group of patients in Stages I or II of SA, compared with TB patients (SA Stage I vs. TB: 8% vs. 0.5% *p* = 0.002, OR = 30.6, CIs 3.63–258.14; SA Stage II vs. TB: 6% vs. 0.5% *p* = 0.004, OR = 22.12, CIs 2.69–181.68; SA Stages I/II vs. TB: 7% vs. 0.5% *p* = 0.002, OR = 25.36, CIs 3.29–195.39). It was accompanied by a less frequent occurrence of the NA1 allele in patients with Stage II of SA, in comparison to TB patients (31.5% vs. 42.5%, *p* = 0.04, OR = 0.63, CIs 0.41–0.97).

This overrepresentation of the SH allele in the initial stages of SA is also visible as an increase in the NA1/SH genotype occurrence in patients with Stages I, II and joint Stage I/II of SA compared with TB (SA Stage I vs. TB: 11% vs. 0%, *p* = 0.01; SA Stage II vs. TB: 8.5% vs. 0%, *p* = 0.02; SA Stage I/II vs. TB: 10% vs. 0%, *p* = 0.01; for all comparisons OR = ∞, CIs 0–∞). In the joint group of SA patients with Stage I or II (I/II) and patients in Stage II of the disease, an increase in the NA2/NA2 genotype was found (vs. TB), comprising of NA2 alleles similar to the SH allele (SA Stage I/II vs. TB: 35% vs. 22%, *p* = 0.03, OR = 1.85, CIs 1.07–3.18; SA stage II vs. TB: 42% vs. 22%, *p* = 0.001, OR = 2.48, CIs 1.33–4.62). Complementary to this, a lower incidence of NA1/NA2 genotype presence was found in SA patients with Stages I, II or I/II of the disease compared with TB patients (SA Stage I vs. TB: 47% vs. 69%, *p* = 0.01, OR = 0.40, CIs 0.20–0.81; SA Stage II vs. TB: 38% vs. 69%, *p* < 0.0001, OR = 0.28, CIs 0.15–0.51; SA stage I/II vs. TB: 42% vs. 69%, *p* < 0.0001, OR = 0.32, CIs 0.19–0.53).

The lower frequency of the NA1/NA2 genotype was found also in SA patients in the more advanced Stages III and III/IV of the disease compared with the TB group (SA Stage III vs. TB: 49% vs. 69%, *p* = 0.03, OR = 0.42, CIs 0.20–0.87; SA Stage III/IV vs. TB: 52% vs. 69%, *p* = 0.02, OR = 0.48, CIs 0.26–0.88). However, it was accompanied, not by an increase in the genotypes made of SH or NA2 alleles, but with a higher frequency of the NA1/NA1 genotype occurrence in patients with Stage III SA, as opposed to TB patients (20% vs. 8%, *p* = 0.05, OR = 2.95, CIs 1.09–7.94).

## 4. Discussion

In our previous studies we have shown changes in the phagocytic activity of blood monocytes and neutrophils in SA and TB patients. We revealed the increased phagocytic activity of monocytes in both disorders, while an increased activity of neutrophils was shown in TB vs. SA [11,26]. The monocytes in SA patients (but not in TB) were resistant to apoptosis and present in high numbers in their blood [27]. We have also shown an increased expression of the FcγRI-III receptors on blood monocytes in SA patients vs. healthy individuals, and an increased fraction of monocytes with FcγRII receptors in TB patients [12,13,14]. The high phagocytic activity of these immune cells in SA, together with an increased expression of Fcγ receptors, responsible for immune complex binding and clearance, should have prevented immunocomplexemia in the blood of our SA patients. However, it did not [8,10]. We have found a high level of immune complexes, circulating in the blood, especially in SA patients with the initial stages of the disease [28]. Therefore, we hypothesised that there was a functional polymorphism of the *FCGR2A, FCGR2B, FCGR2C, FCGR3A* and/or *FCGR3B* genes, encoding Fcγ receptors responsible the for clearance of immune complexes and opsonised pathogens in SA and/or TB patients [14].

We had confirmed our assumptions about the differences in the frequency of *FCGR* gene variants in SA patients and healthy controls, which we have presented in our two previous articles [12,13]. In summary, in the initial stages of SA, Stages I and II, a lower level of 131HH and higher level of the 131HR genotype of *FCGR2A* compared to the more advanced stage of SA, Stage III, had previously been found, [12]. In our previous study, we have also found a decrease in the occurrence of the 57XQ genotype of the *FCGR2C* gene in SA patients in Stages I/II or II of the disease, compared to patients who have developed the more severe stages of SA, Stages III or III/IV [12]. We have also shown previously an increase in the frequency of the 158F and 158FF variants, and a decrease in the 158FV genotype of *FCGR3A* in SA patients in Stage I of the disease vs. healthy controls [13]. There was no difference in the frequency of the *FCGR2B* and *FCGR3B* gene variants between SA patients and healthy individuals [12].

Our current work broadens this analysis greatly. It not only presents for the first time the comparison of *FCGR* polymorphism in TB patients and healthy individuals, but also compares the frequency of the *FCGR* gene variants in SA and TB patients. Thus, the current work aims to find molecular biomarkers to support the differentiation between sarcoidosis and tuberculosis.

In agreement with our previous studies, we have found a significant decrease in the presence of the 57Q allele and 57XQ genotype of the *FCGR2C* gene in SA compared with the TB patient group. We have also found an increase in 158FF and a decrease in the 158FV genotype occurrence of the *FCGR3A* gene, especially in patients with Stage I of SA compared to TB patients. Although in our previous study on genetic predisposition to develop SA we showed no differences between SA patients and healthy controls regarding variants of *FCGR3B* gene, we have shown in this study significant differences between SA and TB patient groups. In the whole group of SA patients, as well as among patients with the initial stages of SA, Stages I and II, we have observed an increased frequency of *FCGR3B* variants (alleles and genotypes) with SH or NA2 alleles, in comparison to TB patients.

Therefore, in the current study we have shown a decrease in the occurrence of *FCGR* gene variants, responsible for more efficient binding of immune complexes in SA versus TB patients. It appeared as a lower presence of the 57Q allele and 57XQ genotype of *FCGR2C*, the 158FV genotype of *FCGR3A*, the NA1 variant and NA1/NA2 genotype of the *FCGR3B* gene (Table 5). We have also shown an increase in the frequency of alleles and genotypes responsible for a less efficient ICs binding in SA compared to TB patients (57X and 57XX of *FCGR2C*, 158FF of *FCGR3A*, SH, NA1/SH, NA2/NA2 of *FCGR3B*). This applied especially to the patients in the initial stages of sarcoidosis, Stages I and II, compared with TB patients.

This is in concordance with our previous studies on the phagocytic activity of neutrophils, which mostly have FcγRIIIb on their surface. Their phagocytic activity was significantly lower in SA patients in comparison to individuals suffering from TB [26].

All of these observations may be associated with a lower ability to bind and clear IgG-based immune complexes and the resulting immunocomplexemia, especially in patients developing the initial, less severe stages of SA compared with TB patients (Table 5) [12,13]. The polymorphism of the *FCGR* genes in patients who developed the initial stages of sarcoidosis, Stages I/II, may cause an aberrant function of Fcγ receptors, located at the surface of immune cells in the peripheral blood of these SA patients. The malfunctioned receptors bind immune complexes with lower affinity and do not clear the immune complexes effectively from circulation via phagocytosis [14]. Therefore, the immune complexes remain in the circulation on a high level. When the immune complexes are not phagocyted effectively, the antigen (bound in ICs) is not effectively processed and presented at the surface of immune cells to trigger immune response as massive as those in more advanced stages of SA (Stages III/IV) and in TB. This lower immune response in the initial stages of SA, Stages I/II, can promote the persistence of an environmental factor causing the disease, e.g., mycobacteria, which can spread its antigens, e.g., Mtb-HSPs, resembling human HSPs. This can subsequently lead to autoimmunisation (via molecular mimicry) and further binding these antigens by IgG to create more immune complexes in the blood of SA patients (reviewed in [29]).

In our previous study, we have revealed a higher frequency of 131HH and lower occurrence of the 131HR genotype of the *FCGR2A* gene in more the advanced stage of SA, Stage III, compared to healthy individuals. We have also found an increased occurrence of 131HH and a decreased occurrence of the 131HR genotype in SA patients with Stages III/IV of the disease in comparison with patients with less severe stages, Stages I/II [12]. We have presented a higher frequency of the 57Q and 57XQ genotypes, as well as a lower occurrence of the 57X allele and the 57XX genotype of *FCGR2C* in SA patients with advanced stages, Stages III and III/IV, compared with healthy individuals and SA patients in the initial stages, Stages I and II [12].

This is in agreement with the fact that we have shown an increase in the 131HH genetic variant of FcγRIIa in patients who have developed more severe stage of SA, Stage III (vs. TB patients). This genetic variant is responsible for more efficient ICs binding by FcγIIa, present in high numbers, especially on monocytes (Table 5). IC binding by this receptor can be followed by the increased phagocytic activity of monocytes, as it was found in our previous study [11]. It can subsequently lead to the processing of the ingested antigen and its presentation at the cell surface to activate a strong immune reaction, present in the advanced stages of SA [14,30].

Therefore, our study showed differences in the genetic background regarding *FCGR* polymorphism, which predisposes the development of sarcoidosis or tuberculosis. Moreover, one can notice differences in the presence of *FCGR* gene variants in the initial or advanced stages of SA, in comparison to TB, which can further support the prognosis of SA development.

An added value of the current study is the comparison of the frequencies of *FCGR* gene variants between TB patients and healthy individuals. This has never been achieved before for people from the Caucasian ethnic group. Additionally, there is only scarce information on the topic about other ethnic groups.

Among TB patients, in comparison to healthy individuals, we have found an increased occurrence of the 57Q allele and 57XQ genotype of *FCGR2C*, leading to an expression of a functional FcγRIIc receptor. We have also found an increase in the NA1/NA2 genotype and a decrease in the SH allele, NA1/SH and NA2/NA2 genotypes of the *FCGR3B* gene in TB patients vs. healthy controls. This points to the expression of a more IC-binding FcγRIIIb receptor in TB patients than in the control group.

The only other study on *FCGR* polymorphism in TB patients was conducted with a Moroccan population sample. In accordance with our findings within the Caucasian ethnic group, no difference was found in the frequency of *FCGR2A* or *FCGR3A* gene variants between TB patients and healthy individuals from the Moroccan population [31]. The cited study did not include an analysis of other *FCGR* genes. Information on *FCGR* polymorphism in TB patients is scarce and does not enable a broader comparison.

Our findings are in accordance with data on the important role of natural killer (NK) cells (possessing FcγRIIc) during the course of tuberculosis in immunocompromised individuals. Activated (e.g., by FcγRIIc) NK cells act towards the inhibition of *M. tuberculosis* growth and inform other immune cells, such as macrophages and monocytes, of the presence of tubercle bacilli [32]. This can be supported by the presented increase in the ability of IC binding by FcγRIIIb receptor in TB patients in comparison to healthy individuals. Neutrophils activated by IC-binding to FcγRIIIb can degranulate with bactericidal enzymes and α-defensins to kill bacilli. Activated neutrophils can also secrete many pro-inflammatory cytokines, e.g., IL-1-β, IL-8 and IFN-γ, in response to mycobacterial infection to inform other immune cells of the threat. They can also engulf bacilli in the process of FcγR-mediated phagocytosis and present mycobacterial antigens on their surface to activate other immune cells in the pursuit of fighting infection [33].

Thus, an increased ability of FcγRIIc and FcγRIIIb to bind immune complexes, including those with mycobacterial antigens, might subsequently translate into a strong inflammatory response, seen in active pulmonary TB, like in our patients.

To conclude, the presented data point to a possibility of supporting the differential diagnosis of SA and TB on the basis of recognition of the genetic variants of *FCGR2C, FCGR3A* and *FCGR3B* genes. Analysis of *FCGR2A* polymorphism can also be helpful in differentiating more severe forms of SA from TB. On the contrary, both in the current study and in our previous analyses of *FCGR* polymorphism in SA patients [34], there were no differences in the frequency of *FCGR2B* alleles or genotypes occurrence in the studied groups. This points to the more important role of regulating FcγRIIa, FcγRIIc, FcγRIIIa and FcγRIIIb activation, rather than FcγRIIb inhibition, during the development of sarcoidosis and tuberculosis.

Therefore, it could be highly beneficial to perform more studies on *FCGR2C/3A/3B* gene polymorphism in SA and TB patients to prove their usefulness as molecular biomarkers during the differential diagnosis of the two highly similar disorders.

Additionally, the presence of higher IC-binding variants of *FCGR2A* and *FCGR3B* genes in individuals with advanced stages of SA vs. TB patients, can help to predict if sarcoidosis will progress to a more severe disorder and serve as a marker of SA prognosis. 

**Table 5:** The influence of *FCGR* gene polymorphism on immunoglobulin G-based immune complex binding by the receptors for Fc fragment of IgG (FcγR).

Location of Receptor	Receptor	Location of aa Change in the Receptor Protein	Gene	Gene Variant Responsible for		**Reference**
				Higher ICs Binding	Lower ICs Binding	
Mo, MΦ, N, less often on E, B, MC	FcγRIIa	second extracellular domain	*FCGR2A*	131H	131R	[14,35,36,37,38]
Almost all types of leukocytes (including B cells and memory CD8^+^ T cells), minority of NK; liver and aorta cells	FcγRIIb	transmembrane domain	*FCGR2B*	232T *	232I *	[14,35,36,38,39,40,41]
NK, less often on Mo, MΦ, N	FcγRIIc	first extracellular domain	*FCGR2C*	57Q	57X (truncated, nonfunctional receptor)	[14,35,36,38,42]
NK, Mo, MΦ, N, T γδ cells, DC, MC, E	FcγRIIIa	second extracellular domain	*FCGR3A*	158V	158F	[14,35,36,37,38]
N, less often on B, after induction on E	FcγRIIIb	first extracellular domain	*FCGR3B*	NA1	NA2, SH	[14,35,36,38,43]

aa—amino acid; ICs—immune complexes; IgG—immunoglobulin G; IgG1/2/3/4—subclasses of IgG; B—basophils; DC—dendritic cells; E—eosinophils; MC—mast cells; Mo—monocytes; MΦ—macrophages; N—neutrophils; NK—natural killer cells; Pl—platelets; * The presence of isoleucine in 232 position of the transmembrane domain of FcγRIIb receptor is associated with a better positioning of the receptor protein in the lipid rafts of the cellular membranes of immune cells in comparison to the presence of threonine in this position. Thus, the FcγRIIb receptor with the 232I variant enables for better regulation (inhibition) of other FcγRs activation.

## Figures and Tables

**Table 1:** Clinical characteristics of patients with sarcoidosis (SA), pulmonary tuberculosis (TB) and healthy volunteers (C). The percentages (%) of individuals with a certain characteristic are given in parentheses.

		SA n = 154 (%)	TB n = 179 (%)	C n = 148 (%)
Age (years)	Mean ± SD	44 ± 13	46 ± 15	42 ± 10
	Median	44	45	40
	Range	21–89	20–80	18–79
Sex	Female	70 (46)	75 (42)	67 (45)
	Male	84 (54)	104 (58)	81 (55)
Smokers		90 (58)	145 (81)	80 (54)
BCG vaccinated		154 (100)	179 (100)	148 (100)
Positive PPD skin test		0	179 (100)	0
Löfgren’s syndrome		28 (18)	0	0
Relapse		0	0	0
Symptoms	Cough	71 (46)	161 (90)	0
	Dyspnea	81 (53)	45 (25)	0
	Increased body temperature	21 (14)	116 (65)	0
	Night sweats	2 (<1)	90 (50)	0

BCG—Bacillus Calmette–Guérin vaccine against tuberculosis; SD—standard deviation; a purified protein derivative (PPD) skin test is a method used to diagnose TB infection.

**Table 2:** Number of patients at specific stages of sarcoidosis (SA) with certain characteristics of the disease. The percentages (%) of individuals with a certain characteristic are given in parentheses.

Stage of SA	I	II	III	IV
Total	38	60	35	21
with Löfgren’s syndrome	13	10	2	0
with the duration of SA > 2 y	14 (37)	42 (70)	35 (100)	21 (100)

y—years.

**Table 3:** Designation of the studied genes and their polymorphism; sequences of the primers used to study it with PCR-SSP method.

Gene Name	NCBI Reference Gene Sequence	Mutation in DNA with dbSNP ID	Amino Acid Change	Primer Names and Sequences for PCR-SSP (5′ to 3′)	Product Length (bp)	Reference
*FCGR2A*	NG_012066.2	A519G, rs1801274	H131R	2A-gene: * TCAAAGTGAAACAACAGCCTGACT 2A-519A: GGAAAATCCCAGAAATTCACACA 2A-519G: GGAAAATCCCAGAAATTCACACG	371	[17]
*FCGR2C*	NG_011982.1	T202C, rs10917661	X57Q	2C-gene: GAGATTCCCATTGTGGACCTACG 2C-202T: GGCTGTGCTGAAACTGGAGACCT 2C-202C: GGCTGTGCTGAAACTGGAGCCAC	124	[18]
*FCGR3A*	NG_009066.1	T559G, rs396991	V158F	3A-gene: AGTTCATCATAATTCTGACTCCT 3A-559T: TGAAGACACATTTCTACTCCCTAA 3A-559G: TGAAGACACATTTCTACTCCCTAC	100	[17]
*FCGR3B*	NG_032926.2	NA1/NA2 differences: C147T, rs447536 G141C, rs403016 A227G, rs448740 G277A, rs428888 G349A, rs2290834 SH/NA2 difference: C266A, rs5030738	L38L R36S N65S D82N V106I A78D	3B-gene: ATGGACTTCTAGCTGCAC 3B-NA1: CAGTGGTTTCACAATGAGAA 3B-NA2/SH: CAATGGTACAGCGTGCTT 3B-SH: TCGAGCTACTTCATTGACGA	NA1: 140 NA2/SH: 219 SH: 102	[19]
*hGH*	NC_000017.11	-	-	hGH-F: CAGTGCCTTCCCAACCATTCCCTTA hGH-R: ATCCACTCACGGATTTCTGTTGTGTTTC	439	[20]
*NF1*	NG_009018.1	-	-	NF1-exon4b-F: CTGTCCCCTAATACTTAATT NF1-exon4b-R: AATACTAGTTTTTGACCCAGT NF1-exon22-F: TCTTTAGCTTCCTACCTAAGAA NF1-exon22-R: AACACACATACACACAAAATGAA	209 262	[21]

bp—base pairs; db SNP ID—identification number in database of single nucleotide polymorphism; F—forward primer; NA1/NA2/SH—variants of *FCGR3B* gene; NCBI—the National Center for Biotechnology Information; PCR-SSP—polymerase chain reaction with sequence-specific primers; R—reverse primer; * primers with ‘gene’ designation are gene-specific, and were coupled with one of the allele-specific primers in a PCR reaction.

**Table 4:** Designation of the polymorphism, sequences of the primers and probes used to study the variants of *FCGR2B* gene (reference gene sequence according to the National Center for Biotechnology Information: NG_023318.2) with the use of a real-time PCR method. Sequences of the primers and probes were designed within the applied biosystems company service.

Mutation in DNA with dbSNP ID	Amino Acid Change	Primer Names and Sequences (5′ to 3′)	Fluorescent Probes	**Product Length (bp)**
T695C, rs1050501	I232T	2B-SNP_F: CCTAGCTCCCAGCTCTTCAC 2B-SNP_R: CCACTACAGCAGCAACAATGG	2B-SNP_695T-VIC: TCACTGGGATTGCTG 2B-SNP_695C-FAM: TCACTGGGACTGCTG	36

bp—base pairs; db SNP ID—identification number in database of single nucleotide polymorphism; F—forward primer; R—reverse primer; VIC and FAM—fluorescent dyes enabling to differentiate between the two variants of *FCGR2B* gene.

## Data Availability

The data presented in this study is available in the current article and Supplementary Materials.

## Supplementary Materials

The following supporting information can be downloaded at: https://www.mdpi.com/article/10.3390/cells12091221/s1, Table S1: Frequency of the occurrence of individual *FCGR2A* alleles in the studied groups and subgroups; Table S2: Frequency of the occurrence of individual *FCGR2A* genotypes in the studied groups and subgroups; Table S3: Frequency of the occurrence of individual *FCGR2B* alleles in the studied groups and subgroups; Table S4: Frequency of the occurrence of individual *FCGR2B* genotypes in the studied groups and subgroups; Table S5: Frequency of the occurrence of individual *FCGR2C* alleles in the studied groups and subgroups; Table S6: Frequency of the occurrence of individual *FCGR2C* genotypes in the studied groups and subgroups; Table S7: Frequency of the occurrence of individual *FCGR3A* alleles in the studied groups and subgroups; Table S8: Frequency of the occurrence of individual *FCGR3A* genotypes in the studied groups and subgroups; Table S9: Frequency of the occurrence of individual *FCGR3B* alleles in the studied groups and subgroups; Table S10: Frequency of the occurrence of individual *FCGR3B* genotypes in the studied groups and subgroups.
