# Supplementary material for: Comparative Analysis of FCGR Gene Polymorphism in Pulmonary Sarcoidosis and Tuberculosis
Source: Cells. 2023 Apr 23;12(9):1221. doi: 10.3390/cells12091221 (PMC10177102; doi:10.3390/cells12091221)
Supplement: Supplementary file 1 [file cells-12-01221-s001.zip › cells-2315010-supplementary.pdf]

Supplementary Materials to article:

Comparative Analysis of *FCGR* Gene Polymorphism in Pulmonary Sarcoidosis and Tuberculosis

Marlena Typiak [1†] & Bartłomiej Rękawiecki [2†], Krzysztof Rębała [3] and Anna Dubaniewicz [2,*]

1 Department of General and Medical Biochemistry, Faculty of Biology, University of Gdansk, 80-308 Gdansk, Poland; marlena.typiak@ug.edu.pl
2 Department of Pulmonology, Medical University of Gdansk, 80-214 Gdansk, Poland
3 Department of Forensic Medicine, Medical University of Gdansk, 80-204 Gdansk, Poland
* Correspondence: aduban@gumed.edu.pl
† These authors contributed equally to this work.

Table S1. Frequency of the occurrence of individual *FCGR2A* alleles in the studied groups and subgroups. Cont. – healthy control; SA – group of patients with sarcoidosis; TB – group of patients with tuberculosis.

Studied group	Frequency of *FCGR2A* alleles [n (%)]	
	**131H**	**131R**
SA total (n = 144)	161 (56)	127 (44)
SA stage I (n = 36)	42 (58)	30 (42)
SA stage II (n = 53)	56 (53)	50 (47)
SA stage III (n = 34)	43 (63)	25 (37)
SA stage IV (n = 21)	20 (49)	22 (51)
TB (n = 179)	199 (56)	159 (44)
Cont. (n = 145)	169 (58)	121 (42)

Table S2. Frequency of the occurrence of individual *FCGR2A* genotypes in the studied groups and subgroups. Cont. – healthy control; SA – group of patients with sarcoidosis; TB – group of patients with tuberculosis.

Studied group	Frequency of *FCGR2A* genotypes [n (%)]		
	**131HH**	**131HR**	**131RR**
SA total (n = 144)	44 (30.5)	73 (51)	27 (18.5)
SA stage I (n = 36)	10 (28)	22 (61)	4 (11)
SA stage II (n = 53)	13 (24)	30 (57)	10 (19)
SA stage III (n = 34)	17 (50)	9 (26)	8 (24)
SA stage IV (n = 21)	4 (19)	12 (57)	5 (24)
TB (n = 179)	53 (30)	93 (52)	33 (18)
Cont. (n = 145)	49 (34)	71 (49)	25 (17)

Table S3. Frequency of the occurrence of individual *FCGR2B* alleles in the studied groups and subgroups. Cont. – healthy control; SA – group of patients with sarcoidosis; TB – group of patients with tuberculosis.

Studied group	Frequency of *FCGR2B* alleles [n (%)]	
	**232I**	**232T**
SA total (n = 134)	233 (87)	35 (13)
SA stage I (n = 30)	52 (87)	8 (13)
SA stage II (n = 49)	86 (88)	12 (12)
SA stage III (n = 34)	58 (85)	10 (15)
SA stage IV (n = 21)	37 (88)	5 (12)
TB (n = 97)	167 (86)	27 (14)
Cont. (n = 101)	173 (85.5)	29 (14.5)

Table S4. Frequency of the occurrence of individual *FCGR2B* genotypes in the studied groups and subgroups. Cont. – healthy control; SA – group of patients with sarcoidosis; TB – group of patients with tuberculosis.

**Studied group**	**Frequency of *FCGR2B* genotypes [n (%)]**		
	**232II**	**232IT**	**232TT**
SA total (n = 134)	100 (74.5)	33 (24.5)	1 (1)
SA stage I (n = 30)	23 (77)	6 (20)	1 (3)
SA stage II (n = 49)	37 (75.5)	12 (24.5)	0
SA stage III (n = 34)	24 (70.5)	10 (29.5)	0
SA stage IV (n = 21)	16 (76)	5 (24)	0
TB (n = 97)	70 (72)	27 (28)	0
Cont. (n = 101)	73 (72)	27 (27)	1 (1)

Table S5. Frequency of the occurrence of individual *FCGR2C* alleles in the studied groups and subgroups. Cont. – healthy control; SA – group of patients with sarcoidosis; TB – group of patients with tuberculosis.

Studied group	Frequency of *FCGR2C* alleles [n (%)]		
	57X	57Q	Gene deletions
SA total (n = 149)	220 (74)	76 (25.5)	1 (0.5)
SA stage I (n = 37)	61 (82)	13 (18)	0
SA stage II (n = 57)	94 (82)	20 (18)	0
SA stage III (n = 34)	43 (63)	25 (37)	0
SA stage IV (n = 21)	22 (52)	18 (43)	1 (5)
TB (n = 179)	187 (52)	163 (45.5)	4 (2.5)
Cont. (n = 148)	257 (87)	39 (13)	0

Table S6. Frequency of the occurrence of individual *FCGR2C* genotypes in the studied groups and subgroups. Cont. – healthy control; SA – group of patients with sarcoidosis; TB – group of patients with tuberculosis.

**Studied group**	**Frequency of *FCGR2C* genotypes [n (%)]**			
	**57XX**	**57XQ**	**57QQ**	**Gene deletions**
SA total (n = 149)	78 (52)	64 (43)	6 (4)	1 (1)
SA stage I (n = 37)	25 (68)	11 (30)	1 (2)	0
SA stage II (n = 57)	40 (70)	14 (25)	3 (5)	0
SA stage III (n = 34)	9 (26)	25 (74)	0	0
SA stage IV (n = 21)	4 (19)	14 (67)	2 (9)	1 (5)
TB (n = 179)	17 (9)	153 (85)	5 (3)	4 (3)
Cont. (n = 148)	112 (76)	33 (22)	3 (2)	0

Table S7. Frequency of the occurrence of individual *FCGR3A* alleles in the studied groups and subgroups. Cont. – healthy control; SA – group of patients with sarcoidosis; TB – group of patients with tuberculosis.

Studied group	Frequency of *FCGR3A* alleles [n (%)]	
	**158F**	**158V**
SA total (n = 120)	142 (59)	98 (41)
SA stage I (n = 23)	33 (72)	13 (28)
SA stage II (n = 54)	52 (48)	56 (52)
SA stage III (n = 23)	31 (67)	15 (33)
SA stage IV (n = 20)	26 (65)	14 (35)
TB (n = 179)	193 (54)	165 (46)
Cont. (n = 148)	167 (56)	129 (44)

Table S8. Frequency of the occurrence of individual *FCGR3A* genotypes in the studied groups and subgroups. Cont. – healthy control; SA – group of patients with sarcoidosis; TB – group of patients with tuberculosis.

**Studied group**	**Frequency of *FCGR3A* genotypes [n (%)]**		
	**158FF**	**158FV**	**158VV**
SA total (n = 120)	47 (39)	48 (40)	25 (21)
SA stage I (n = 23)	13 (57)	7 (30)	3 (13)
SA stage II (n = 54)	13 (24)	26 (48)	15 (28)
SA stage III (n = 23)	12 (52)	7 (30.5)	4 (17.5)
SA stage IV (n = 20)	9 (45)	8 (40)	3 (15)
TB (n = 179)	49 (27)	95 (53)	35 (20)
Cont. (n = 148)	42 (28)	83 (56)	23 (16)

Table S9. Frequency of the occurrence of individual *FCGR3B* alleles in the studied groups and subgroups. Cont. – healthy control; SA – group of patients with sarcoidosis; TB – group of patients with tuberculosis.

Studied group	Frequency of *FCGR3B* alleles [n (%)]		
	NA1	NA2	SH
SA total (n = 154)	111 (36)	184 (60)	13 (4)
SA stage I (n = 38)	32 (42)	38 (50)	6 (8)
SA stage II (n = 60)	38 (31.5)	75 (62.5)	7 (6)
SA stage III (n = 35)	25 (36)	45 (64)	0
SA stage IV (n = 21)	16 (38)	26 (62)	0
TB (n = 179)	152 (42.5)	205 (57)	1 (0.5)
Cont. (n = 148)	113 (38)	168 (56.5)	15 (5.5)

Table S10. Frequency of the occurrence of individual *FCGR3B* genotypes in the studied groups and subgroups. Cont. – healthy control; SA – group of patients with sarcoidosis; TB – group of patients with tuberculosis.

**Studied group**	**Frequency of *FCGR3B* genotypes [n (%)]**					
	**NA1/NA1**	**NA1/NA2**	**NA1/SH**	**NA2/NA2**	**NA2/SH**	**SH/SH**
SA total (n = 154)	19 (12)	70 (45.5)	9 (6)	52 (34)	4 (2.5)	0
SA stage I (n = 38)	5 (13)	18 (47)	4 (11)	9 (24)	2 (5)	0
SA stage II (n = 60)	5 (8.5)	23 (38)	5 (8.5)	25 (42)	2 (3)	0
SA stage III (n = 35)	7 (20)	17 (49)	0	11 (31)	0	0
SA stage IV (n = 21)	2 (10)	12 (57)	0	7 (33)	0	0
TB (n = 179)	14 (8)	124 (69)	0	40 (22)	1 (1)	0
Cont. (n = 148)	21 (14)	57 (39)	14 (9)	55 (37)	1 (1)	0
